# T/L-type calcium channel blocker reduces the composite ranking of relative risk according to new KDIGO guidelines in patients with chronic kidney disease

**DOI:** 10.1186/1471-2369-14-135

**Published:** 2013-07-01

**Authors:** Masanori Abe, Kazuyoshi Okada, Hiroko Suzuki, Yoshinori Yoshida, Masayoshi Soma

**Affiliations:** 1 Division of Nephrology, Hypertension and Endocrinology, Department of Internal Medicine, Nihon University School of Medicine, Tokyo, Japan; 2 Division of General Medicine, Department of Internal Medicine, Nihon University School of Medicine, Tokyo, Japan

**Keywords:** Benidipine, Calcium channel blocker, Kidney Disease: Improving Global Outcomes (KDIGO), T-type calcium channel

## Abstract

**Background:**

Recently, the Kidney Disease: Improving Global Outcomes (KDIGO) group recommended that patients with chronic kidney disease (CKD) be assigned according to stage and composite relative risk on the basis of glomerular filtration rate (GFR) and albuminuria criteria. The aim of this post-hoc analysis was to investigate the effects of add-on therapy with calcium channel blockers (CCBs) on changes in the composite ranking of relative risk according to KDIGO guidelines. Benidipine, an L- and T-type CCB, and amlodipine, an L-type CCB to angiotensin II receptor blocker (ARB), were examined.

**Methods:**

Patients with blood pressure (BP) > 130/80 mmHg, an estimated GFR (eGFR) of 30–90 mL/min/1.73 m^2^, and albuminuria > 30 mg/gCr, despite treatment with the maximum recommended dose of ARB, were randomly assigned to two groups. Each group received one of two treatments: 2 mg benidipine daily, increased to 8 mg daily (n = 52), or 2.5 mg amlodipine daily, increased to 10 mg daily (n = 52).

**Results:**

After 6 months of treatment, a significant and comparable reduction in systolic and diastolic BP was observed in both groups. The eGFR was significantly decreased in the amlodipine group, but there was no significant change in the benidipine group. The decrease in albuminuria in the benidipine group was significantly lower than in the amlodipine group. The composite ranking of relative risk according to the new KDIGO guidelines was significantly improved in the benidipine group; however, no significant change was noted in the amlodipine group. Moreover, significantly fewer cases in the benidipine group than the amlodipine group showed a reduced risk category score.

**Conclusion:**

The present post-hoc analysis showed that compared to amlodipine benidipine results in a greater reduction in albuminuria accompanied by an improved composite ranking of relative risk according to the KDIGO CKD severity classification.

**Trial registration:**

Trial registration Number: UMIN000002644

## Background

Chronic kidney disease (CKD) progressively increases the risk of cardiovascular disease and end-stage renal disease (ESRD) in line with its severity [1]. In 2002, the Kidney Disease Outcomes Quality Initiative(KDOQI)organization published a guideline providing diagnosis and classification of CKD into five stages according to severity using the glomerular filtration rate (GFR) as the main criterion [2]. Recent studies have shown that individuals with a GFR ≤45 ml/min/1.73 m^2^are atincreased risk compared with those with a higher GFR [1,3-5]. The presence of proteinuria also increases cardio-renal events significantly [1]. As a result, in 2009, the Kidney Disease: Improving Global Outcomes (KDIGO) group recommended that individuals be classified according to proteinuria stage as well as GFR stage [1]. The diagnostic criteria for CKD remained unchanged, but the new KDIGO guideline divided stage 3 (30 < GFR < 60 ml/min/1.73 m^2^) into the following 2 substages: 3a (GFR, 45 to < 60) and 3b (GFR, 30 to < 45). In addition, clinicians and researchers were advised to categorize patients using a “heat map” generated by the composite ranking of relative risk.

Renin-angiotensin system blockade with angiotensin-converting enzyme inhibitors and angiotensin II type-1 receptor blockers (ARBs) are respectively considered the most effective pharmacological approaches for renoprotection, reducing proteinuria more effectively than other antihypertensive drugs [6,7]. Current guidelines recommend blood pressure (BP) levels <130/80 mmHg in patients with CKD [8]. However, this reduction is difficult to achieve in CKD, and a combination of two or more antihypertensive agents is needed in more than 60% of patients [9]. In line with recent molecular biological studies, Ca channels are now classified into five subtypes: L, T, N, P/Q, and R, according to their location and function [10,11], with three types of Ca channel blocker (CCB): L-, T-, and N-types, currently in clinical use. Both L- and T-type Ca channels are present in afferent arterioles, whereas only T-type channels are present in efferent arterioles. Benidipine has been shown to block both L- and T-type channels, causing dilatation of both efferent and afferent arterioles [12], and is therefore expected to be more advantageous than amlodipine, an L-type CCB, in progressing renal dysfunction and decreasing albuminuria in patients with CKD [13-15].

We previously reported the renoprotective effects of benidipine compared with amlodipine in patients with CKD [13]. The main finding showed that compared to amlodipine benidipine enhanced the maximum recommended dose of ARBs (80 mg telmisartan daily and 40 mg olmesartan daily, respectively) while reducing albuminuria and plasma aldosterone levels over a 6-month study period, independent of its BP-lowering effect. The aim of this post-hoc analysis was to investigate the effects of benidipine and amlodipine on changes in the composite ranking of relative risk according to the new KDIGO guidelines.

## Methods

We previously conducted a 6-month, single-center, prospective, randomized, open-label clinical trial [13], designed to compare the effects of benidipine and amlodipine on blood pressure (BP), estimated glomerular filtration rate (eGFR), and urinary albumin excretion ratio in hypertensive and albuminuric patients with CKD already receiving the maximum recommended dose of ARBs. In the present study, conducted between June 2009 and May 2010, post-hoc analysis was performed to compare the effects of benidipine and amlodipine on changes in the composite ranking of relative risk in the same population according to the 2009 KDIGO guidelines. All study participants provided written informed consent, and the trial protocol was approved by the Research Review Board of Nerima Hikarigaoka Hospital, Nihon University School of Medicine and conducted in accordance with the Declaration of Helsinki. This study was not supported by any grants. Subjects were followed for 6 months.

Inclusion criteria were (1) hypertension (systolic/diastolic BP ≥ 130/80 mmHg measured in the sitting position on at least two separate clinic visits), (2) stage 2–3 CKD (eGFR 30–90 mL/min/1.73 m^2^) with albuminuria (urinary albumin/creatinine (Cr) ratio ≥ 30 mg/g · Cr; average of two consecutive measurements taken during a 4-week period before treatment), and (3) treatment with the maximum recommended ARB dose (80 mg telmisartan daily or 40 mg olmesartan daily) for at least 8 weeks prior to the study.

Exclusion criteria were (1) age <20 years and >80 years; (2) hypertensive emergency; (3) history of severe heart failure, angina, myocardial infarction, or stroke within 6 months prior to the start of the trial; (4) previous treatment with steroids or immunosuppressants; (5) renovascular hypertension, as determined by renal Doppler ultrasonography before enrollment in the study, or endocrine hypertension; and (6) severe diabetes mellitus, resulting in hospitalization because of extremely high plasma glucose, or with complications such as diabetic ketoacidosis.

Subjects were randomly assigned to two groups prior to the start of the study. An independent investigator with no previous knowledge of the subjects before commencement of the trial monitored randomization of the order of entry of the subjects. Dynamic balancing randomization was carried out on the basis of age, gender, serum Cr (sCr) levels and the urinary albumin/Cr ratio measured at the time of registration, and the presence or absence of diabetic nephropathy. Thus, we ensured that no significant differences existed between the baseline characteristics of each group. The details of the assignment were then given to four independent investigators. Patients then received one of the following two treatment regimens: 2 mg benidipine daily, increased to a daily dose of 8 mg (benidipine group), or 2.5 mg amlodipine daily, increased to a daily dose of 10 mg (amlodipine group).

BP was measured at the outpatient clinic at fixed times after administration of medication according to the Japanese Society of Hypertension 2009 guidelines [8]. Measurements were performed in duplicate ever month using a sphygmomanometer (Nippon Colin, Tokyo, Japan) with the patient in a sitting position after a 5-min rest. Patients, particularly those with dietary restrictions, were given guidance on how to maintain their diet. Doses of ARBs and ACE inhibitors were not altered during the study period.

The target BP level was <130/80 mmHg. During the study period, patients were administered a combination drug therapy that included other conventional antihypertensive agents administered at baseline. Withdrawal of treatment was considered in patients who developed an allergy/intolerance to benidipine or amlodipine during the study period, experienced a hypertensive emergency, or developed any other condition or received another therapy that, in the opinion of the investigators, might pose a risk or confound the results of the study. Furthermore, patients administered additional antihypertensive medication (other than ARBs or CCBs) to achieve the target BP, when benidipine or amlodipine failed to do so, were excluded.

All parameters used to monitor the effects of the drugs were evaluated once a month during a 6-month treatment period. Serum samples were assayed for Cr in a central laboratory (Central Laboratory; SRL Co, Tokyo, Japan) by means of the enzymatic Cr assay method using a Japan electron Cr auto-analyzer, model JCA-BM8060 (JEOL Ltd., Tokyo, Japan) and enzyme solution (Preauto-S CRE-L; Sekisui Medical Co., Ltd., Tokyo, Japan). The sCr values obtained in the central laboratory were compared with the standard reference material (SRM914a, The National Institute of Standards and Technology, Gaithersburg, USA) by using a calibration panel of 50 samples. To assess urinary albumin excretion, we measured urinary concentrations of albumin and Cr (albumin/Cr ratio) in an early-morning spot urine sample. Urinary albumin was measured using the immunoturbidimetric assay. Treatment compliance and safety variables were monitored at each visit to our hospital. Glomerular filtration rate was estimated using the modified final recommendation equation for Japanese patients of the Japanese Society of Nephrology-CKD Initiatives (JSN-CKDI), since eGFR values obtained by this method are more accurate for Japanese patients with CKD [16]. The following formula was used: eGFR (mL/min/1.73 m^2^) = 194 × sCr^–1.094^ × age^–0.287^ (× 0.739 for women).

**Figure 1:**
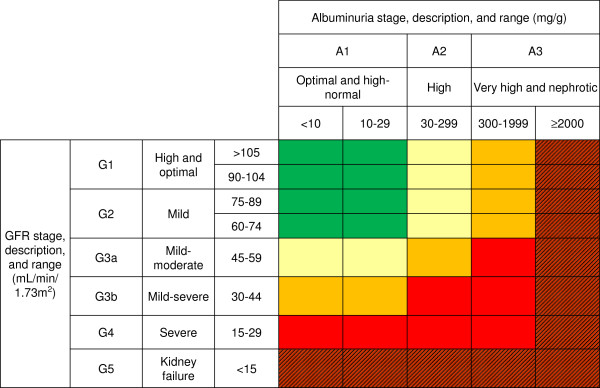
New CKD classification of relative risk according toGFR and albuminuria (KDIGO 2009).

The composite ranking of relative risk by GFR and albuminuria levels was calculated according to the 2009 KDIGO recommendations using the following equations: Risk Category No CKD G1A1, G2A1; Moderate risk G1A2, G2A2, G3aA1; High risk G1A3, G2A3, G3aA2, G3bA1; Very-high risk G3aA3, G3bA2-3, all G4, and all G5 (Figure 1) [1]. Changes in the composite ranking before and six months after CCB treatment were assessed based on this category in each group. “Risk reduction” was defined as an improved risk category after amlodipine or benidipine treatment. On the other hand, “Risk increase” was defined as a worsened risk category after treatment.

### Statistical analysis

Data were analyzed on the basis of randomly assigned groups, regardless of participants’ subsequent medication (intention-to-treat analysis), and expressed as the mean ± SEM. Baseline characteristics of the patients were compared between treatment groups using the unpaired *t*-test and chi-squared test. Mean values of each group were then compared using the unpaired *t*-test. The Student’s *t*-test was applied to determine the effect of treatment on BP, heart rate, and urinary albumin/Cr ratio. eGFR time course data within group swere analyzed by repeated-measures analysis of variance (ANOVA), while changes between the two groups were analyzed by two-way ANOVA followed by Dunnett’s test. Correlations were determined using the Spearman rank correlation test. Changes in risk categories were calculated according to the following score allocation: No CKD, 0 points; moderate risk, 1 point; high risk, 2 points; and very-high risk, 3 points, then changes in scores compared between baseline and the end of treatment using the Mann–Whitney U test. Changes in the proportion showing a “Risk reduction” and “Risk increase” between groups were compared using the chi-squared test. Statistical analyses were performed with SPSS Version 19.0 (SPSS Inc., IL, USA) with significance set at P < 0.05.

## Results

### Study population and baseline characteristics

A total of 104 subjects were enrolled in this study and randomly allocated to the benidipine (n = 52) or amlodipine group (n = 52). Baseline characteristics and medications at baseline are shown in Table 1. No significant differences were observed between groups with regard to baseline characteristics or the number of patients with diabetic nephropathy. Adequate BP control had not been achieved at baseline in any of the enrolled patients. During treatment, two subjects from each group were excluded because additional antihypertensive medications (benidipine group: furosemide, n = 1, thiazide diuretics, n = 1; amlodipine group: furosemide, n = 2) were required to achieve the target BP or improve edema. Therefore, 100 subjects completed the trial. There was no significant difference in the proportion of risk categories between groups at baseline.

**Table 1:** Patient characteristics and medication details

	**Amlodipine group**	**Benidipine group**	**P value**
	**(n = 52)**	**(n = 52)**	
Sex (male/female)	30/22	30/22	-
Age (years)	67.5 ± 1.5	67.3 ± 1.4	0.97
Diabetes Mellitus (n(%))	23(44.2)	24(46.1)	0.84
Cause of CKD (n(%))			
Diabetic nephropathy	23(44.2)	24(46.1)	0.97
Chronic glomerulonephritis	15(28.8)	14(27.0)	0.82
Hypertensive nephrosclerosis	14(27.0)	14(27.0)	-
Hemoglobin A1c (%) (for diabetes)	6.48 ± 0.15	6.43 ± 0.11	0.93
Systolic blood pressure (mmHg)	145 ± 1.0	144 ± 0.9	0.46
Diastolic blood pressure (mmHg)	81 ± 1.3	82 ± 1.4	0.73
Sodium (mEq/L)	140 ± 0.3	140 ± 0.4	0.97
Potassium (mEq/L)	4.4 ± 0.1	4.5 ± 0.1	0.17
Baseline therapy (n(%))			
Details of ARB			
Telmisartan 80 mg daily	28(53.8)	29(55.8)	0.84
Olmesartan 40 mg daily	24(46.2)	23(44.2)	0.84
ACE inhibitors	4(7.7)	3(5.8)	0.69
Diuretics	7(13.4)	6(11.5)	0.76
β-blockers	4(7.7)	5(9.6)	0.73
α-blockers	4(7.7)	4(7.7)	-

*CKD* Chronic kidney disease, *ACE* angiotensin-converting enzyme, *ARB*, angiotensin receptor blocker, Mean ± SEM using the unpaired *t*-test or chi-squared test.

### BP-lowering effect

The final doses of benidipine and amlodipine for the two groups were 6.3 ± 0.3 and 5.4 ± 0.4 mg per day, respectively. Systolic and diastolic BPs were significantly decreased in both groups by the end of the study compared with baseline (both P < 0.001). Systolic BP was decreased by -17.1 ± 0.9 mmHg and -16.3 ± 0.8 mmHg in the amlodipine and benidipine groups, respectively, but the difference was not significant. Diastolic BP was also decreased by - 9.1 ± 1.1 mmHg and -7.8 ± 0.9 mmHg, respectively, but again the difference was not significant. Heart rate at the end of the study was significantly reduced in the benidipine group compared with baseline (benidipine group: 75.1 ± 1.4 to 73.7 ± 1.3 beats per min, P < 0.001; amlodipine group: 74.5 ± 1.7 to 74.8 ± 1.6 beats per min, not significant). A significant difference was also observed in the change in heart rate between groups (amlodipine group:+0.3 ± 0.6 beats per min; benidipine group: -1.4 ± 0.4 beats per min; P =0.038).

### Renoprotective effects

**Figure 2:**
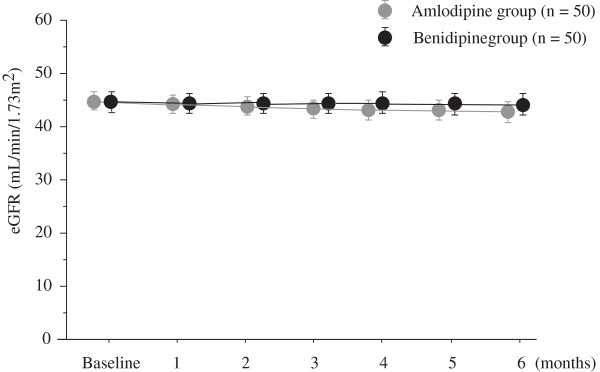
**Changes in estimated glomerular filtration rate between the two treatment groups during the study period.** Grey circles: amlodipine group, black circles: benidipine group, mean ± SEM.

As shown in Table 2, there was a significant reduction in the urinary albumin/Cr ratio in the benidipine group (baseline 175.5 ± 22.4 mg/g·Cr to end of the study 120.6 ± 13.6 mg/g·Cr; P < 0.001), but not the amlodipine group (baseline 173.2 ± 18.7 mg/g·Cr to the end 194.1 ± 34.6 mg/g·Cr). Figure 2 shows the changes in eGFR during the 6-month treatment period in both groups. Changes in eGFR were significantly decreased in the amlodipine group (44.7 ± 1.7 mL/min/1.73 m^2^ at baseline to 42.7 ± 1.9 mL/min/1.73 m^2^ at the end of the study; P = 0.006), but no significant changes were observed in the benidipine group (44.6 ± 1.9 mL/min/1.73 m^2^ at baseline to 44.3 ± 2.1 mL/min/1.73 m^2^ at the end of the study; P = 0.519). As shown in Table 2, during the study period eGFR reduced by - 2.0 ± 0.7 mL/min/1.73 m^2^ and -0.2 ± 0.7 mL/min/1.73 m^2^ in the amlodipine and benidipine groups, respectively (P = 0.032).There was no significant correlation between the changes in albuminuria and eGFR in either group (amlodipine group; r = 0.081, P = 0.575, benidipine group; r = -0.127, P = 0.379).

**Table 2:** Changes in the urinary albumin/Cr ratio and eGFR

	**Amlodipine group**	**Benidipine group**	**P value**
	**(n = 50)**	**(n = 50)**	**(Amlodipine vs. Benidipine)**
Urinary albumin (mg/g • Cr)			
Pre	173.2 ± 18.7	175.5 ± 22.4	0.965
Post	194.1 ± 34.6	120.6 ± 13.6	0.564
⊿Urinary albumin	20.8 ± 32.1	-54.9 ± 13.7	0.08
P value (pre vs. post)	0.774	<0.001	
eGFR (mL/min/1.73 m^2^)			
Pre	44.7 ± 1.7	44.6 ± 1.9	0.937
Post	42.7 ± 1.9	44.3 ± 2.1	0.051
⊿eGFR	-2.0 ± 0.7	-0.2 ± 0.7	0.032
P value (pre vs. post)	0.006	0.519	

Mean ± SEM using the paired t-test or unpaired t-test.

### Composite ranking of relative risk

**Figure 3:**
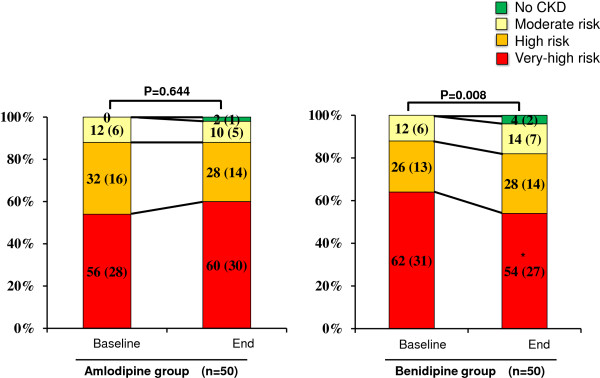
**Changes in CKD severity according to KDIGO 2009 categories between baseline and after CCB treatment in the two treatment groups.** Numbers in bars indicate % (n). * P < 0.05 vs. at baseline.

**Figure 4:**
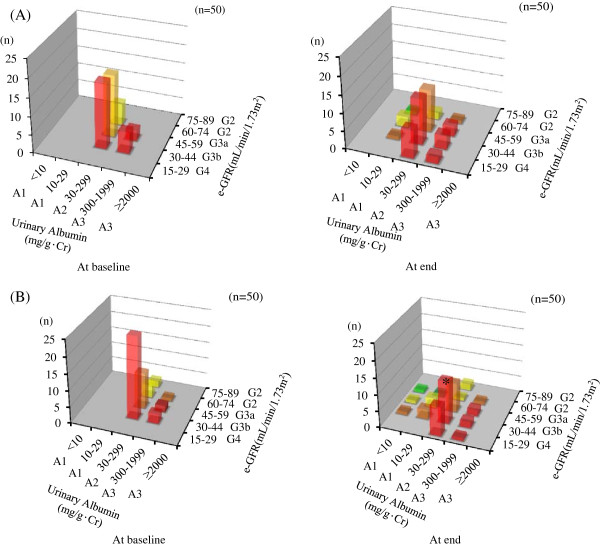
**Changes in details of the KDIGO 2009 categories at baseline and after CCB treatment.** In **(A)** the amlodipine group and **(B)** the benidipine group. * P < 0.001 vs. at baseline.

**Figure 5:**
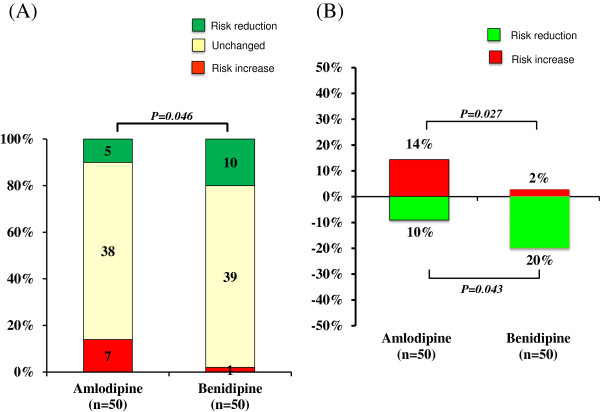
**Difference in CKD severity changes between the two treatment groups. (A)** Changes in risk scores in the two treatment groups. Numbers in bars indicate the number of patients. **(B)** Comparison of the proportion with “Risk reduction” and “Risk increase” between the two treatment groups.

Changes in risk categories in each group are shown in Figure 3. Although there was no significant change in the proportion of each category or relative risk scores after amlodipine treatment, benidipine treatment significantly decreased relative risk scores (P = 0.008). Furthermore, the proportion of those in the very high risk category was significantly decreased after benidipine treatment. Figure 4 shows the change in each category between baseline and the end of treatment in both groups. As shown in Figure 4B, the number of patients in the G3bA2 category was significantly reduced by benidipine treatment, although there were no changes in any category in the amlodipine group (Figure 4A). As shown in Figure 5A, a significant difference was noted in the change in risk scores between the amlodipine and benidipine groups. Furthermore, although there was no significant difference in the proportion of unchanged categories, the proportion showing “Risk reduction” was significantly higher while that of “Risk increase” was significantly lower in the benidipine group compared to the amlodipine group. As shown in Figure 5B, “Risk reduction” was found in 5 (10%) and 10 cases (20%) in the amlodipine and benidipine groups, respectively (P = 0.043). Moreover, although “Risk increase” was observed in 7 cases (14%) in the amlodipine group, only 1 case (2%) was found in the benidipine group (P = 0.027).

## Discussion

Recent studies have revealed that proteinuria and albuminuria are risk factors for both end-stage renal disease and cardiovascular disease (CVD) in CKD patients [2,17]. Accordingly, reductions in proteinuria and albuminuria are associated with a reducing trend in renal death and cardiovascular events [18,19]. These findings extend the novel concept that high albuminuria/proteinuria alone should be a target for reducing hard endpoints, as in established treatments for high BP, high blood glucose, and high LDL-cholesterol. Thus, albuminuria/proteinuria reduction is one of the most important surrogate goals of hypertension treatment as it reduces both renal death and CVD. It has also been reported in post-hoc analysis of RENAAL and IDNT trials that a dual approach targeting both BP and albuminuria is important in improving cardiovascular outcomes [20]. Furthermore, post-hoc analysis of the IDNT trial using eGFR as the principal outcome measure confirmed that ARB irbesartan significantly slows the long-term rate of decline in eGFR, resulting in delayed progression towards ESRD by at least 33%. This finding was explained by reductions in BP and proteinuria [21]. Previous studies have demonstrated that the reducing effects of benidipine on albuminuria/proteinuria are due to efferent arteriolar dilation followed by attenuation of glomerular hypertension [14,15,22,23]. In the present study, the respective add-on effects of benidipine and amlodipine on albuminuria reduction were demonstrated in 100 CKD patients on top of the ARBs telmisartan and olmesartan. Compared with amlodipine, albuminuria reduction was obtained with benidipine combined with ARB. Moreover, benidipine reduced albuminuria while maintaining the eGFR, and as a result, more patients showed a “Risk reduction” after benidipine compared to amlodipine treatment.

Meta-analysis of the effects of CCB on major adverse cardiovascular events (MACE) in vasospastic angina patients revealed that the hazard ratio for the occurrence of MACE was significantly lower in those treated with benidipine than other CCBs [24,25]. Moreover, benidipine is reportedly more selective towards coronary artery smooth muscle cells that other CCBs amlodipine, nifedipine, and diltiazem [26,27]. This higher selectivity of benidipine towards coronary arteries might be due not only to its inhibitory effect on coronary artery spasm, but also its improved prognostic effect. Furthermore, this higher affinity of benidipine might also be related to its long-lasting effects, independent of the blood concentration [28,29]. The beneficial prognostic effect of benidipine compared with other CCBs was therefore noted, suggesting involvement of vasculoprotective effects, but not anti-vasospastic effects. Indeed, it was recently shown that benidipine improves vascular endothelial function, including flow-mediated dilation, pulse wave velocity, and the augmentation index in patients with hypertension [30-32]. Moreover, benidipine was also shown to reduce myocardial infarction (MI) size by increasing nitric oxide production and inhibiting free radical production in a rabbit model of MI [33].

The mechanisms behind the association between albuminuria and CVD remain largely unknown and are the focus of intensive research and debate [34,35]. It has been suggested that albuminuria not only reflects glomerular damage, but also serves as a sensitive indicator of generalized endothelial dysfunction and capillary vasculopathy that leads to penetration of atherosclerotic lipoproteins into the arterial wall [36-38]. Studies have shown that albuminuria is associated with endothelial dysfunction in the systemic circulation [29]. As endothelial and vascular damage become advanced, more and more glomeruli are injured, resulting in a substantial amount of albuminuria and reduced GFR. Therefore, albuminuria, which indicates the presence of advanced glomerular as well as systemic vascular lesions, is a very high risk factor for both renal and cardiovascular events in subjects with CKD [35]. Thus, this suggests that the reduction in albuminuria by benidipine is due not only to the efferent arteriolar dilation effect but also the improvement in endothelial dysfunction. Since benidipine has vasculoprotective effects on both the coronary artery and the glomerulus through improvements in vascular endothelial function, it is expected to reduce both renal events and cardiovascular disease. However, determining whether this is indeed the case based on the reduced risk category according to the new KDIGO classification requires further long-term investigations.

Although the risk categories in the KDIGO guidelines were formed using pooled outcome data from multiple populations through application of the Modification of Diet in Renal Disease (MDRD) Study equation, it has been reported that the Chronic Kidney Disease Epidemiology Collaboration (CKD-EPI) equation more accurately estimates GFR using the same variables, especially at higher GFR [39]. The CKD-EPI equation is a better predictor of risk than the MDRD Study equation in CKD cohorts as well as in cohorts with higher eGFR [39]. However, CKD-EPI equations were developed in mostly Caucasian and African American populations. A previous study revealed that eGFR values obtained using CKD-EPI equations with sCr were significantly higher than the actual GFR in Japanese subjects [40]. When the eGFR was calculated using a coefficient-modified CKD-EPI equation based on sCr (0.813 × CKD-EPI) in the present study [41,42], changes in eGFR were 43.6 ± 2.6 mL/min/1.73 m^2^ at baseline to 41.2 ± 2.6 mL/min/1.73 m^2^ at the end of the study (P = 0.006) in the amlodipine group and 44.0 ± 2.8 mL/min/1.73 m^2^ at baseline to 43.9 ± 3.3 mL/min/1.73 m^2^ at the end of the study (P = 0.945) in the benidipine group. No significant differences were noted between the Japanese GFR equation and the CKD-EPI equation when calculating the proportion of each category or relative risk score. Therefore, we could also use the CKD-EPI equation to assess the composite ranking of relative risk on the basis of GFR values. When the bias, precision, and accuracy of the GFR equations were compared in Japanese subjects stratified by measured GFR, Japanese GFR equations were revealed to be effective for patients with a GFR < 60 mL/min/1.73 m^2^, compared with the coefficient-modified CKD-EPI equations [40]. Furthermore, in a study using the Japanese GFR equation, reduced eGFR was independently associated with incident CVD events in Japanese patients with type-2 diabetic nephropathy and patients with non-diabetic CKD [43,44]. In the present study, since the mean eGFR at baseline was 44.6 ± 1.9 mL/min/1.73 m^2^, we assessed the changes in eGFR using the Japanese GFR equation.

Despite the present findings, our study is limited by the relatively small sample size and the short period of treatment. Moreover, the changes in sCr levels were too small for adequate evaluation of the influence of CCB therapy. It has been reported that there is insufficient evidence to assume that a reduction in albuminuria levels will lead to an improvement in clinical outcomes such as progression to ESRD, a CVD event, or death [45]. Additional studies are therefore necessary to more firmly establish the validity of changes in albuminuria as a surrogate for kidney disease progression. Furthermore, long-term investigations are also necessary to accurately assess the preventive renal and cardiovascular effects of benidipine therapy in patients with CKD. Moreover, to assess the changes in risk categories of the KDIGO classification that precisely reflect prognosis, requirements for renal replacement therapy and other renal or cardiovascular events should be considered endpoints.

## Conclusions

The present post-hoc analysis showed that compared to amlodipine, an L-type CCB, benidipine, an L- and T-type CCB, results in a greater reduction in albuminuria and improved composite ranking of relative risk according to the 2009 KDIGO CKD severity classification in patients with CKD. These effects of benidipine seem to make the drug more advantageous in terms of the progression of renal outcome and prevention of cardiovascular events in patients with hypertensive CKD. However, further studies are needed to determine whether long-term use of benidipine can actually reduce renal events and cardiovascular morbidity in patients with CKD.

## Competing interests

The authors declare no conflict of interest.

## Author’s contributions

MA conceived of the study and participated in its design, advised throughout the study and at final approval, and helped draft the manuscript. OK, HS and YY participated in its design and coordination, drafted the manuscript and performed statistical analysis. MS reviewed the study design and revised the manuscript. All authors read and approved the final manuscript.

## Pre-publication history

The pre-publication history for this paper can be accessed here:

http://www.biomedcentral.com/1471-2369/14/135/prepub
